# B Cell Immunity in Solid Organ Transplantation

**DOI:** 10.3389/fimmu.2016.00686

**Published:** 2017-01-10

**Authors:** Gonca E. Karahan, Frans H. J. Claas, Sebastiaan Heidt

**Affiliations:** ^1^Department of Immunohaematology and Blood Transfusion, Leiden University Medical Center, Leiden, Netherlands

**Keywords:** HLA, donor-specific antibodies, antigen presentation, cognate T–B interactions, memory B cells, rejection

## Abstract

The contribution of B cells to alloimmune responses is gradually being understood in more detail. We now know that B cells can perpetuate alloimmune responses in multiple ways: (i) differentiation into antibody-producing plasma cells; (ii) sustaining long-term humoral immune memory; (iii) serving as antigen-presenting cells; (iv) organizing the formation of tertiary lymphoid organs; and (v) secreting pro- as well as anti-inflammatory cytokines. The cross-talk between B cells and T cells in the course of immune responses forms the basis of these diverse functions. In the setting of organ transplantation, focus has gradually shifted from T cells to B cells, with an increased notion that B cells are more than mere precursors of antibody-producing plasma cells. In this review, we discuss the various roles of B cells in the generation of alloimmune responses beyond antibody production, as well as possibilities to specifically interfere with B cell activation.

## Introduction

**Figure 1:**
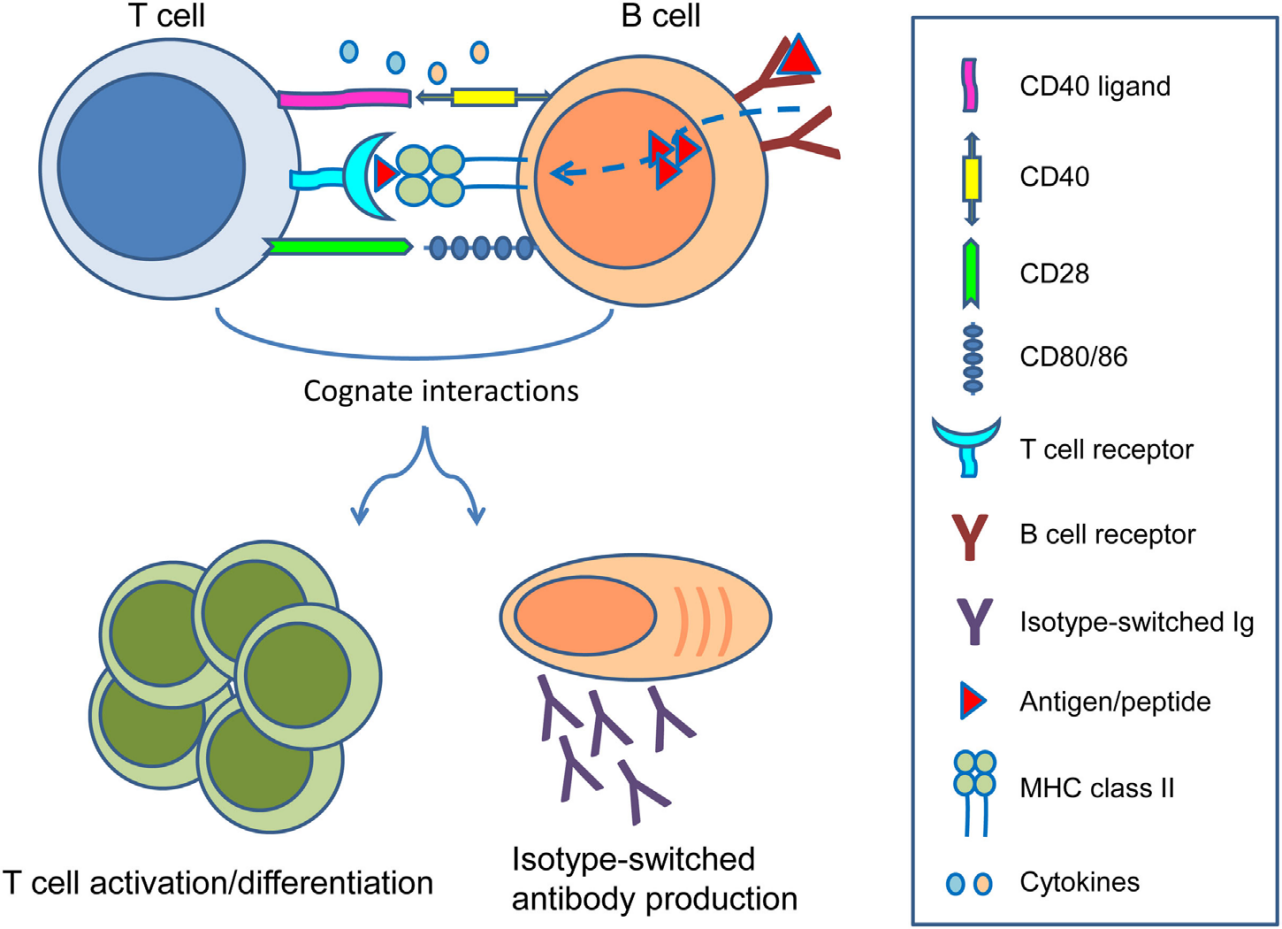
**Reciprocal interactions between T cells and B cells**. Following B cell receptor-mediated uptake of protein antigens, activated B cells process and present antigenic peptides in the context of major histocompatibility complex (MHC) class II on their surface to cognate T cells that recognize the MHC–peptide complex through their T cell receptor. Ligation of CD40 ligand and CD28 on T cells to CD40 and CD80/86 on B cells, as well as production of several cytokines enable differentiation of both B cells and T cells into effector and memory subsets. While B cells can become isotype-switched antibody-producing plasma cells and memory B cells, T cells can become activated as effectors or differentiate into memory T cells to sustain cellular immune responses.

In the setting of organ transplantation, B cells are primarily known for their ability to differentiate into long-lived plasma cells producing high affinity, class-switched alloantibodies. The detrimental role of pre-existing donor-reactive antibodies at time of transplantation was already described in the 60s of the previous century in the form of hyperacute rejection (1). With the introduction of the complement-dependent cytotoxicity crossmatch assay by Terasaki and colleagues, the problem of hyperacute rejection was largely eliminated (2, 3). In the decades that followed focus shifted toward T cells and the prevention of cellular rejection. As a consequence, many drugs have been developed to successfully keep T cell immunity in check (4). With T cells largely under control, it is now clear that B cells remain important as precursors of antibody-producing plasma cells. However, B cells also give rise to humoral immune memory in the form of memory B cells, process and present alloantigens to T cells, are involved in ectopic lymphoid follicle formation, and modulate T cell responses by secreting cytokines. Reciprocal cognate interactions between T cells and B cells play key roles in the generation of alloimmune responses (5) (Figure 1).

In order to understand how B cells contribute to adaptive immune responses, we will first summarize the basics of human B cell development. Afterward, we will focus on the various roles of B cells in the setting of solid organ transplantation by antibody production, alloantigen presentation to T cells, intragraft tertiary lymphoid organ formation, as well as immune regulation. Finally, we will discuss new venues in interfering with B cell activation.

## Generation of Humoral Immune Responses in Secondary Lymphoid Organs

### B Cell Development in Bone Marrow

B cells are crucial components of the humoral immune response. They participate in eradication of pathogens by their ability to differentiate into antibody-producing plasma cells, thereby propagating long-term serological immune memory. B cell development encompasses a programed set of events that initiate in primary lymphoid organs, which advances to a functional maturation stage in secondary lymphoid organs. Development and survival of B cells depend on the cell surface expression of a functional antigen receptor, namely, the B cell receptor (BCR), which is a membrane-bound immunoglobulin (Ig) molecule in complex with Ig α/β heterodimer signaling molecules (6). In order to generate a functional BCR capable of recognizing a broad range of antigens but not self, the gene segments encoding the BCR go through rearrangements in the bone marrow, by the assembly of variable (V), diversity (D), and joining (J) gene segments at both Ig heavy and light chain loci *via* DNA recombination (7). Newly formed B cells that express autoreactive BCRs are modified either by receptor editing or deleted by apoptosis. Upon completion of receptor editing, immature B cells with an intact BCR on their cell surface leave the bone marrow as transitional B cells to further continue maturation in the peripheral circulation and secondary lymphoid organs (8).

Modifications of the BCR proceed in germinal centers (GCs) at later stages of B cell differentiation during T cell-dependent immune responses as discussed below. While certain B cell subsets respond to polysaccharide antigens such as non-self blood group antigens by producing natural antibodies independent of T cell help, responses to protein antigens [e.g., human leukocyte antigen (HLA)] develop in the presence of T cell help. Since alloimmune responses are generally directed at protein antigens, we will focus on T cell-dependent follicular B cell responses.

### B Cell Activation in Secondary Lymphoid Organs

Secondary lymphoid organs are located at strategic sites throughout the body and provide the proper environment for T and B cells to come into contact with antigen and interact with each other. Both aspects are essential for the generation of antibody responses. In lymph nodes, B cells form follicles in the cortex just beneath the subcapsular sinus (SCS) of the lymphatic vessel, while T cells are localized in the paracortex adjacent to B cell follicles. The paracortex contains high endothelial venules through which lymphocytes and dendritic cells enter the lymph node (9). Immature naïve B cells continuously circulate through the peripheral blood, lymph, and enter secondary lymphoid organs in order to gain access into B cell follicles where they can complete their maturation and receive further survival signals. These naïve B cells home to secondary lymphoid organs through chemokines secreted by a network of stromal and follicular dendritic cells (FDC) (10–12). If a B cell does not encounter its specific antigen it detaches from FDC, leaves the lymph node *via* efferent lymphatics, and continues to recirculate between peripheral blood and secondary lymphoid organs (13).

Mature naïve B cells can become activated when their BCR engages an intact antigen inside or outside primary B cell follicles. While follicular B cells can recognize antigen presented on the surface of FDC, small soluble antigens can quickly diffuse from SCS into B cell follicles and can directly be recognized by BCRs. Large antigens such as immune complexes and viruses can be transported to B cell follicles by specialized CD169^+^ macrophages resident at SCS. These macrophages lack phagocytosis ability and can present the antigen in its intact form to B cells (14). The immunological synapse between antigen-presenting cell (APC) and BCR initiates downstream signaling events and rearrangements of the B cell cytoskeleton. Subsequently, B cells that have acquired and processed antigen move toward the boundaries of T and B cell zones to survey for cognate T cell help. CD4^+^ T cells in interfollicular and paracortical T cell zones initially interact with cognate antigen-presenting dendritic cells and subsequently increase their ability to migrate to B cell follicles.

A mature naïve B cell requires two signals to become activated: the first signal is received through the engagement of its BCR with cognate antigen and the second through cognate interaction with CD4^+^ T cells, termed as follicular helper T cells (T_FH_). Upon receiving T cell help at the T–B cell border, B cells can either differentiate into short-lived extrafollicular plasmablasts that produce low-affinity IgM antibodies or can proceed to go through GC reactions.

### GC Reactions

Repositioning of antigen-activated T and B cells from the T–B cell zone back to the follicle initiates the GC reaction. During this transient reaction, B cells start to proliferate and consequently trigger the egress of naïve, circulating B cells from the primary follicle. The follicle resolves into light and dark zones harboring B cells at various levels of cell division. Although the exact mechanisms that define the fate of B cells in GC are not entirely understood, signaling through the BCR and interactions with T_FH_ are known to be essential for their survival and differentiation into long-lived plasma cells and memory B cells. B cells present antigen to T_FH_ in GCs for the second time during the course of the humoral immune response. GC B cells with high-affinity BCR appear to be most efficient at antigen uptake, processing, and presentation to T_FH_ cells as well as being more prone to survival than those with low-affinity BCR. Ligation of peptide/major histocompatibility complex (MHC) class II, CD40, and CD80/86 on B cells with the TCR, CD40L, and CD28 on T cells, respectively, in the presence of cytokines such as IL-2, IL-4, IL-5, and IL-21 appear to be crucial (15–17). The activated B cells undergo clonal expansion, class switch recombination from IgM to IgG, IgA, or IgE and acquire somatic hypermutations in the variable region of their BCR (18, 19). Affinity-driven selection enables further proliferation and differentiation of B cells with high-affinity BCR into long-lived plasma cells and memory B cells (20). While long-lived plasma cells preferentially home to the bone marrow, memory B cells remain quiescent until re-encounter with antigen and recirculate between secondary lymphoid organs and the peripheral blood (21, 22). Generation of rapid antibody responses following antigen re-challenge requires efficient antigen presentation by memory B cells to cognate memory T_FH_. Upon receipt of this T cell help, memory B cells rapidly differentiate into plasma cells and produce high levels of antigen-specific, mainly IgG type of antibodies.

## Why are B Cells Important in Solid Organ Transplantation?

Solid organ transplantation is a life-saving treatment option for patients with end-stage organ failure. The level of genetic disparities at HLA class I and II loci between donor and recipient, as well as the ability of the recipient’s immune system to respond determine the strength of the immune response to an allograft (23–25). Immune responses directed toward mismatched HLA evoke both the cellular and the humoral arm of the adaptive immune system (26, 27). To prevent immunological rejection of the allograft, patients receive life-long immunosuppressive treatment. Currently available immunosuppressive regimens are centered on T cells and have been successful in curtailing acute cellular rejection. Successful treatment of cellular rejection by targeting T cells with immunosuppressive drugs have reduced acute rejection rates and hence improved short-term graft survival. It is clear that these drugs are insufficient in controlling humoral immune responses since antibody-mediated rejection (ABMR) is the leading cause of chronic allograft failure (28, 29). A growing body of evidence suggests that B cells play essential roles in alloimmunity besides mediating humoral immune responses. Understanding the various functions of B cells and the delicate balance between different B cell subsets may facilitate advances in B cell-targeting immunosuppressive drug development and eventually direct toward understanding the mechanisms involved in allograft tolerance.

## Significance of Antibody Responses in Solid Organ Transplantation

Antibodies binding to mismatched HLA (or non-HLA) molecules on donor endothelial cells initiate a set of signaling events leading to recruitment of effector cells to the graft endothelium through complement-dependent and -independent pathways. This process results in graft thrombosis and eventually a decline in allograft function. Clinical studies have shown that both pre-existence and *de novo* production of IgG donor-specific antibodies (DSA) are strongly associated with acute and chronic allograft injury in kidney, heart, lung, and to some extent, liver transplantation (29–34). On the contrary, studies on IgM and IgA DSA did not reveal any isolated effect of these isotypes on allograft outcome unless they were co-existent with IgG antibodies (35, 36). This indicates that the above described GC response needs to be active for pathological antibody response to occur in the setting of organ transplantation.

In accordance with several earlier studies, Loupy et al. found in a large-scale retrospective study on renal transplant recipients that patients developing DSA after transplantation have inferior 5-year graft survival rates compared to those without DSA (37). Among those patients with *de novo* DSA, the capability to fix complement was associated with more severe lesions including microvascular inflammation and C4d deposition. In a recent study, Lefaucheur et al. investigated the role of complement fixation of HLA-DSA in a cohort of 635 kidney transplant recipients (38). The authors categorized patients into three groups: ABMR-free, acute ABMR, and subclinical ABMR. They found that whereas ABMR-free patients most prominently had IgG1^+^ DSA lacking C1q fixing capacity, patients with acute ABMR most frequently showed IgG3^+^ DSA, which was associated with microvascular inflammation, C4d deposition in peritubular capillaries, and inferior graft survival. Interestingly, patients classified as having subclinical ABMR showed IgG2^+^ and IgG4^+^ DSA and had predominantly chronic lesions. Results from this study highlight the divergence between acute complement-dependent and chronic complement-independent roles for HLA-specific antibodies in mediating different types of allograft injury.

While circulating antibodies are mainly produced by long-lived plasma cells residing in the bone marrow, local alloantibody production within intragraft tertiary lymphoid organs has also been described (39). Thaunat et al. demonstrated the presence of alloantibodies in supernatants of renal cortex tissue cultures, suggestive for local antibody production within the graft. Comparison of HLA antibody specificities and strength of the antibody response revealed differences in serum and supernatant samples from the same patient (39). Several studies have shown the presence of DSA eluted either from core needle biopsy samples or explanted renal tissue of patients with failed allografts, which may be due to absorbance of circulating alloantibodies but may also be pointing toward local production (40–42). Huibers et al. found DSA in lysates of coronary arteries of heart allograft autopsies harboring ectopic lymphoid structures. Interestingly, DSA and non-DSA found in the graft and serum at the time of autopsy were directed only against HLA class II (43). A recent study by Milango et al. showed the presence of DSA in both serum and graft eluates at the time of nephrectomy in the absence of immunosuppressive treatment. Although HLA-C and -DP mismatches between the recipients and donors were not analyzed, 80% of HLA antibody specificities were found to be directed at mismatched donor epitopes both for HLA classes I and II (44).

Currently available methods to detect serum HLA (discussed elsewhere in this issue of *Frontiers in Immunology*) do not provide any information on the magnitude of HLA-specific memory B cells (45). As described above, these memory B cells can rapidly differentiate into antibody-secreting cells upon re-challenge. Memory B cells exert this rapid function upon re-encounter with the immunizing HLA or in response to a non-specific innate stimuli due to their lower activation threshold and constitutive toll-like receptor expression (46–48). Several reports have shown the presence of additional HLA antibody specificities that are not detected in serum but in the culture supernatants of polyclonally activated peripheral blood B cells from kidney transplant recipients with a history of sensitization (49, 50). Therefore, studying donor-specific B cell responses in the transplant setting is certainly of importance, and several recently developed techniques allow to do so (51–57).

## A Role for B Cells in Antigen Presentation to Alloreactive T Cells

Expression of high levels of MHC class II and costimulatory molecules on activated B cells, their capacity to take up antigens by their BCR, and ability to clonally expand make B cells also extremely potent APC (58–64). Nonetheless, the APC function of B cells in transplantation setting was initially neglected among others due to murine studies reporting efficient CD4^+^ T cell priming in B cell-deficient mice transplanted with skin or cardiac allografts (65–67). However, it turned out that the developmental absence of B cells may have triggered non-B cell APC to deviate T cell responses toward a Th1 phenotype, thereby potentiating allograft rejection (68).

In order to assess the role of B cells as APC to alloreactive T cells in the transplant setting, Noorchashm et al. generated bone marrow chimeric mice lacking either MHC class II or the MHC class II peptide loading machinery, specifically in B cells (69). Both of these chimeras showed prolonged cardiac allograft survival compared to wild-type controls, which experienced early T cell-mediated rejection. These results indicate that antigen presentation by B cells is involved in T cell-mediated rejection. However, although the authors observed impaired IgG alloantibody production in addition to a decreased CD4^+^ T cell division rate, these experiments did not formally answer the question whether B cells are required for T cell differentiation into effector or memory subsets. This question was addressed by Ng et al. in an allogeneic skin transplantation model using B cell-deficient (μMT) mice. Whereas similar numbers of IFN-γ producing CD4^+^ and CD8^+^ T cells compared to wild type were found early after transplantation (effector phase), at a later stage (memory phase), μMT mice showed decreased numbers of alloreactive IFN-γ^+^ T cells (70). These data suggest that memory T cell development is dependent on the interaction with B cells. While these studies provided evidence for the contribution of B cells to antigen presentation and T cell differentiation, the impact of alloantibodies on transplant outcome was not formally excluded. It appears that both alloantibodies and B cell-dependent T cell activation are important since Burns and colleagues showed that the enhanced T cell-mediated rejection of murine cardiac allografts upon re-challenge is caused by a combined effect of alloantibodies and memory B cell-dependent activation of T cells (71).

In clinical kidney transplantation, the possible role for B cells as APC in T cell-mediated rejection mainly comes from studies on renal biopsies. A landmark study by Sarwal and colleagues showed dense B cell clusters in biopsies of acute cellular rejections that did not correlate with C4d deposition but were associated with steroid resistance and inferior graft survival (72). Since then, several groups confirmed the correlation of graft-infiltrating CD20^+^ B cell clusters with steroid-resistant acute cellular rejection and poor graft survival (73–75), whereas other investigators did not find any prognostic significance of these intragraft B cell clusters neither for treatment sensitivity nor for transplant outcome (76–78). Remarkably, CD20^+^ B cell clusters were mainly present in cases of T cell-mediated rejections without any association to ABMR, which is suggestive for a significant role of B cells other than antibody production (72–75). Indeed, intragraft CD20^+^ B cells have been shown to display an activated, mature phenotype as shown by CD79a and HLA-DR expression and are often found in close proximity to CD4^+^ T cells (75). In an elegant study using cell distance mapping, ICOS^+^CXCR4^+^ F_TH_-like cells were found in close proximity to B cells in both T cell-mediated or mixed cellular rejection, thereby strongly supporting the concept of antigen presentation by these B cells to alloreactive T cells (79).

## B Cells in Tertiary Lymphoid Organs of Chronically Rejected Allografts

Ectopic lymphoid organs resemble canonical secondary lymphoid organs regarding their T and B cell compartmentalization and interaction with dendritic cells, as well as the utilization of chemokine-mediated signaling pathways. By contrast, they display impaired lymphatic drainage and therefore trap the antigen leading to continuous exposure of immune cells to the antigen. *De novo* formation of lymphoid-like structures as a result of persistent antigen exposure at sites of chronic infection or inflammation in non-lymphoid organs has been described in both autoimmunity and cancer (80, 81).

Upon organ transplantation, an environment containing persisting antigen similar to an autoreactive milieu is created and as a result can lead to tertiary lymphoid organ formation (82). Kerjaschki et al. demonstrated proliferating T cells (75%) and B cells (25%) in nodular infiltrates in close proximity with lymphatic vessels in explanted kidney allografts (83). Similarly, Thaunat et al. described the presence of lymphoid neogenesis in virtually all allografts explanted due to chronic rejection (39, 84). B cells in these explants were organized into nodules reminiscent of either primary or secondary B cell follicles. Relatively high expression of genes characteristic for GCs were observed in renal secondary B cell follicles indicating a highly activated phenotype for graft-infiltrating B cells (39, 85). Furthermore, local B cell proliferation, a characteristic for the GC response, occurs as shown by Ki67 positivity and clonality of infiltrating B cells (83–85). In tertiary lymphoid organs, graft-infiltrating B cells might be contributing to lymphoid angiogenesis by prominent expression of vascular endothelial growth factor-A (86). Organization of the lymphoid infiltrates in the form of ectopic GCs may lead to containment of the alloimmune response within the graft. The aforementioned absence of DSA in circulation or discrepancies in specificities or strength of locally produced and circulating HLA antibodies supports this hypothesis (39, 84). It is possible that the infiltrates observed during acute T cell-mediated rejection may represent an early stage of tertiary lymphoid organ development.

## B Cells as Immune Regulators

In addition to their roles in immune activation, (subsets of) B cells may also have regulatory function (87). Several groups have reported B cells with regulatory properties in controlling autoimmunity and inflammation (87–90). A complicating factor in studying regulatory B cells (Bregs) is the lack of a unique marker to define these cells. This has resulted in a wide range of B cell subsets to be identified as Bregs with the ability to secrete IL-10, IL-35, or TGF-β (91–93). In mice, a T cell costimulatory molecule termed as T cell Ig domain and mucin domain (TIM1) was found to be useful for identifying IL-10-producing Bregs (94). In humans, two main subsets of B cells enriched for Bregs have been described: CD24^hi^CD38^hi^ transitional B cells (89) and CD24^hi^CD27^+^ B10 cells (95). Whereas IL-10, IL-35, and TGF-β have all been described as effector molecules of Bregs, in the setting of transplantation, the main focus has been on IL-10-producing B cells.

In transplantation, regulatory functions of B cells have mainly been investigated in murine models of allograft tolerance. Ding et al., using a mouse model of islet transplantation, demonstrated that TIM1 may also have functional properties in Breg development. They observed prolonged allograft survival in mice treated with an agonistic anti-TIM1 antibody compared to untreated mice (94). Interestingly, in mice depleted of B cells before transplantation, anti-TIM1 treatment accelerated allograft rejection, indicating an important role for B cells in TIM1-mediated tolerance. Transfer of TIM1^+^ B cells into untreated recipients of islets led to prolonged allograft survival. This regulatory effect was defective in TIM1^+^ B cells, showing the dependency of B cells on IL-10 for their regulatory capacity. Shortly after, Lee et al. reported 100% long-term islet allograft survival in mice treated with a combination of anti-CD45RB and TIM1 (96). They demonstrated prompt rejection of islet allografts if regulatory T cells (Tregs) were depleted before transplantation, implying that Bregs require an interaction with Tregs to induce tolerance. Furthermore, Le Texier et al. have shown the presence of intragraft IgM^+^ B cells in rats with cardiac allograft tolerance compared to the presence of IgG^+^ B cells in allografts showing chronic rejection (97), suggestive for a restriction in B cell activation in the tolerant group. To demonstrate that tolerance was (at least partially) caused by B cells, the authors performed adoptive transfer of splenic B cells from tolerant rats to show allograft tolerance in these secondary mice.

A hint toward a role for B cells in clinical transplantation tolerance came from studies identifying B cell signatures in operationally tolerant kidney transplant recipients who were immunosuppression-free for at least 1 year with stable graft function (98–100). Microarray analyses on peripheral blood revealed 22 B cell-specific genes that were enriched in tolerant patients compared to those with stable graft function. Furthermore, the CD20 transcript was found to be the only marker higher in urine sediments of tolerant patients. Indeed, three genes (*IGKV4-1, IGLL1*, and *IGKVID-13*) encoding Ig kappa and lambda light chains in the course of B cell differentiation were shown to be predictive of operationally tolerant patients (98). In an accompanying study, six highly overexpressed genes were identified in tolerant patients (*CD79B, TCL1A, SH2D1B, MS4A1, FCRL1*, and *FCRL2*) that were associated with B cell-related pathways (99). Interestingly, expression of *CD79B, MS4A1*, and *TCL1A* has been shown to be significantly downregulated in renal transplant recipients with acute rejection (101, 102).

Tolerant patients showed increased peripheral blood B cell numbers and a redistribution of B cell subsets toward a naïve (IgM^+^IgD^+^CD27^−^) and transitional (CD24^hi^CD38^hi^) phenotype with increased expression of IL-10, compared to patients with stable graft function under immunosuppressive treatment (98, 99). The findings on IL-10-competent transitional B cells are in line with the definition of Bregs as described by Blair et al. (89). Pallier and colleagues confirmed the elevated peripheral blood B cell numbers and found that B cells with a memory phenotype (IgD^−^CD38^−/+^CD27^+^) were increased (103). Whether these are the B10 cells as described by the group of Tedder remains to be established (95). Compared to patients with stable graft function, the majority of the operationally tolerant patients do not have circulating DSA and have a lower frequency of CD38^+^CD138^+^ plasma cells in the peripheral blood (98, 99, 103). In order to determine whether there was a defect in tolerant patients in generating humoral immune responses, Chesneau et al. polyclonally activated purified B cells from operationally tolerant patients *in vitro*. Polyclonally activated B cells proliferated and produced normal levels of IgM and IgG, accompanied by increased levels of IL-10 compared to those with stable graft function (104). In order to asses the inhibitory role of polyclonally activated B cells of tolerant patients on autologous CD4^+^CD25^−^ T cells, Chesneau et al. blocked IL-10, TGF-β, and granzyme B in a T–B cell co-culture system and found that only granzyme inhibitors affected the suppressive effects of B cells (105). However, antigen specificity, a prerequisite for immune regulation, has yet to be demonstrated.

## Effects of Immunosuppressive Treatments on B Cells

In the current practice of kidney transplantation, standard triple immunosuppressive regimen consists of a calcineurin inhibitor (tacrolimus or cyclosporine), a purine analog (mycophenolic acid-MPA), and corticosteriods as maintenance therapy in addition to a non-depleting anti-CD25 monoclonal antibody as the induction agent (106). Since these agents exert their effects preferentially on T cells, they may abrogate humoral immune responses indirectly by inhibiting the T cell help (107), although some of these also have a direct effect on B cells (108, 109). Drugs specifically interfering with humoral immunity can be classified into several groups: drugs that deplete B cells from the circulation, those that interfere with T–B cell interaction, drugs targeting B cell survival signals, and drugs interfering with antibody production or effector function.

Current therapies for (highly) sensitized patients are primarily focused on removal of antibodies before transplantation by plasmapheresis, intravenous immunoglobulins, or immunoadsorption (110). Addition of rituximab, a humanized murine CD20 antibody which depletes circulating CD20^+^ B cells, to desensitization protocols resulted in improved outcomes in ABO-incompatible transplantation (111–113). Surprisingly, when rituximab was administered to non-sensitized patients as induction therapy, a higher rate of acute rejection was observed compared to controls (114). In addition to its application in treatment of ABMR (115), administration of rituximab led to successful treatment of steroid-resistant acute cellular rejections (116) and resolution of B cell infiltrates in graft (117–120). However, in patients experiencing chronic allograft dysfunction, rituximab treatment was ineffective in resolution of tertiary lymphoid organs despite the successful depletion of circulating B cells (121). Kamburova et al. showed long-lasting B cell depletion in patients receiving rituximab as induction agent with repopulating B cells mainly consisting of transitional B cells (122). Similar results were obtained when patients were treated with alemtuzumab, an anti-CD52 monoclonal antibody (123, 124). Although polyclonal activation of purified B cells did not reveal a difference in proliferation or IgM-producing cells, a significant decrease in IgG-producing cells was observed (123).

Another way of attenuating B cell responses can be achieved by blocking the critical costimulatory pathways between T and B cells. A recent study by Chen et al. in a mouse model of cardiac allograft transplantation showed that costimulation blockade with a high-affinity CTLA-4Ig (belatacept) inhibited memory B cell responses and DSA formation, thereby leading to prolonged graft survival (125). By blocking both CD28–CD80/86 (belatacept) and CD40–CD40L (2C10R4) pathways in a non-human primate model of ABMR, Kim et al. showed a decrease in clonal B cell expansion in GCs (126). Combined blockade led to reduced IL-21 production and was strongly associated with reduced DSA levels. Importantly, results of a large phase 3 trial confirmed the efficacy of belatacept in the clinical setting (127). This study revealed a reduction of DSA in the belatacept-treated group with a significant reduced risk of graft loss and death compared to the cyclosporine-treated group.

Several studies have shown increased serum levels of B cell-activating factor (BAFF) following treatment with depleting agents in kidney transplant recipients (128, 129), possibly due to a lack of BAFF consuming B cells. BAFF has a critical role in promoting survival, maturation, and activation of B cells, as well as maintaining self-tolerance (130). High levels of BAFF have been described in the setting of autoimmunity, and it is conceivable that high BAFF levels could also influence alloimmunity. Indeed, elevated serum BAFF levels were associated with increased risk of developing DSA and ABMR in the setting of kidney transplantation (131–133). Blockade of BAFF and/or the related molecule called a proliferation-inducing ligand (APRIL) may be an additional tool to downregulate humoral alloimmune responses as was suggested by the prolonged survival of cardiac allografts in BAFF-deficient mice (134). Also, in a non-human primate ABMR model, BAFF/APRIL blockade (atacicept) was able to prevent *de novo* DSA production (135).

Plasma cells are responsible for the continuous production of antibodies and therefore have a high proteasomal activity. Proteasome inhibitors, such as bortezomib, are effective for the treatment of plasma cell malignancies (136). Bortezomib has been used to treat ABMR and diminish DSA production in sensitized transplant recipients (137–140). However, the inhibitory capacity of proteasome inhibitors is not limited to plasma cells as also naïve and memory B cell proliferation can be affected (141). Therefore, antibody production through plasma cells, as well as the various effects of B cells, may be dampened by proteasome inhibition.

## Conclusion and Remarks

B cells contribute to acute and chronic allograft rejection processes by producing DSA. More recently, other functions have been attributed to B cells that may also influence the alloimmune response, such as antigen presentation to T cells, formation of tertiary lymphoid organs, or secretion of regulatory cytokines.

Considering that one-third of the patients on the kidney waiting lists are sensitized as a result of previous exposure to allogeneic HLA, memory B cells and their effector functions may play central roles in prospective transplantation outcome of these patients. Upon re-challenge, HLA-specific memory B cells generated during primary immune responses can promptly become high-affinity DSA-producing plasma cells and may serve as potent APC by their high expression of HLA-DR and costimulatory molecules. In conclusion, a variety of B cell populations with different functions may affect the alloimmune response after transplantation. Future therapies targeting B cells should take into consideration these different functions and the consequence that a simple depletion of all B cells will also interfere in the beneficial effects of certain B cell subpopulations.

## Author Contributions

GK, FC, and SH designed the outline and wrote the manuscript.

## Conflict of Interest Statement

The authors declare that the research was conducted in the absence of any commercial or financial relationships that could be construed as a potential conflict of interest.

## Abbreviations

ABMR, antibody-mediated rejection; APC, antigen-presenting cell; APRIL, a proliferation-inducing ligand; BAFF, B cell-activating factor; BCR, B cell receptor; Bregs, regulatory B cells; CDC, complement-dependent cytotoxicity; CTLA4, cytotoxic T lymphocyte-associated protein 4; DSA, donor-specific antibody; FDC, follicular dendritic cells; GC, germinal center; HEV, high endothelial venules; HLA, human leukocyte antigens; Ig, immunoglobulin; MHC, major histocompatibility complex; MPA, mycophenolic acid; PRA, panel reactive antibodies; SCS, subcapsular sinus; T_FH_, follicular T helper cells; Tregs, regulatory T cells.

## References

[1] Kissmeyer-Nielsen F Olsen S Petersen V Fjeldborg O Hyperacute rejection of kidney allografts, associated with pre-existing humoral antibodies against donor cells. Lancet (1966) 2(7465):662–5. 10.1016/S0140-6736(66)92829-7 4162350

[2] Terasaki PI McClelland JD Microdroplet assay of human serum cytotoxins. Nature (1964) 204:998–1000. 10.1038/204998b0 14248725

[3] Patel R Terasaki PI Significance of the positive crossmatch test in kidney transplantation. N Engl J Med (1969) 280(14):735–9. 10.1056/NEJM196904032801401 4886455

[4] Halloran PF Immunosuppressive drugs for kidney transplantation. N Engl J Med (2004) 351(26):2715–29. 10.1056/NEJMra033540 15616206

[5] Wood KJ Goto R. Mechanisms of rejection: current perspectives. Transplantation (2012) 93(1):1–10. 10.1097/TP.0b013e31823cab44 22138818

[6] Reth M Antigen receptor tail clue. Nature (1989) 338(6214):383–4. 10.1038/338383b0 2927501

[7] Granato A Chen Y Wesemann DR. Primary immunoglobulin repertoire development: time and space matter. Curr Opin Immunol (2015) 33:126–31. 10.1016/j.coi.2015.02.011 25797714 PMC4414491

[8] Berkowska MA Driessen GJ Bikos V Grosserichter-Wagener C Stamatopoulos K Cerutti A Human memory B cells originate from three distinct germinal center-dependent and -independent maturation pathways. Blood (2011) 118(8):2150–8. 10.1182/blood-2011-04-345579 21690558 PMC3342861

[9] Fu YX Chaplin DD. Development and maturation of secondary lymphoid tissues. Annu Rev Immunol (1999) 17:399–433. 10.1146/annurev.immunol.17.1.399 10358764

[10] Cyster JG Ansel KM Reif K Ekland EH Hyman PL Tang HL Follicular stromal cells and lymphocyte homing to follicles. Immunol Rev (2000) 176:181–93. 10.1034/j.1600-065X.2000.00618.x 11043777

[11] Katakai T Suto H Sugai M Gonda H Togawa A Suematsu S Organizer-like reticular stromal cell layer common to adult secondary lymphoid organs. J Immunol (2008) 181(9):6189–200. 10.4049/jimmunol.181.9.6189 18941209

[12] Reif K Ekland EH Ohl L Nakano H Lipp M Forster R Balanced responsiveness to chemoattractants from adjacent zones determines B-cell position. Nature (2002) 416(6876):94–9. 10.1038/416094a 11882900

[13] Schwab SR Cyster JG. Finding a way out: lymphocyte egress from lymphoid organs. Nat Immunol (2007) 8(12):1295–301. 10.1038/ni1545 18026082

[14] Gaya M Castello A Montaner B Rogers N Reis e Sousa C Bruckbauer A Host response. Inflammation-induced disruption of SCS macrophages impairs B cell responses to secondary infection. Science (2015) 347(6222):667–72. 10.1126/science.aaa1300 25657250

[15] Batista FD Iber D Neuberger MS B cells acquire antigen from target cells after synapse formation. Nature (2001) 411(6836):489–94. 10.1038/35078099 11373683

[16] Cannons JL Qi H Lu KT Dutta M Gomez-Rodriguez J Cheng J Optimal germinal center responses require a multistage T cell:B cell adhesion process involving integrins, SLAM-associated protein, and CD84. Immunity (2010) 32(2):253–65. 10.1016/j.immuni.2010.01.010 20153220 PMC2830297

[17] Qi H Cannons JL Klauschen F Schwartzberg PL Germain RN SAP-controlled T-B cell interactions underlie germinal centre formation. Nature (2008) 455(7214):764–9. 10.1038/nature07345 18843362 PMC2652134

[18] McHeyzer-Williams LJ McHeyzer-Williams MG. Antigen-specific memory B cell development. Annu Rev Immunol (2005) 23:487–513. 10.1146/annurev.immunol.23.021704.115732 15771579

[19] Tarlinton DM. Evolution in miniature: selection, survival and distribution of antigen reactive cells in the germinal centre. Immunol Cell Biol (2008) 86(2):133–8. 10.1038/sj.icb.7100148 18180800

[20] Crotty S Ahmed R. Immunological memory in humans. Semin Immunol (2004) 16(3):197–203. 10.1016/j.smim.2004.02.008 15130504

[21] Kunkel EJ Butcher EC. Plasma-cell homing. Nat Rev Immunol (2003) 3(10):822–9. 10.1038/nri1203 14523388

[22] Agematsu K Hokibara S Nagumo H Komiyama A CD27: a memory B-cell marker. Immunol Today (2000) 21(5):204–6. 10.1016/S0167-5699(00)01605-4 10782048

[23] Claas FH van Rood JJ. The hyperimmunized patient: from sensitization toward transplantation. Transpl Int (1988) 1(2):53–7. 10.1111/j.1432-2277.1988.tb01783.x 3076380

[24] Duquesnoy RJ HLA epitope based matching for transplantation. Transpl Immunol (2014) 31(1):1–6. 10.1016/j.trim.2014.04.004 24769079

[25] Lin CM Gill RG. Direct and indirect allograft recognition: pathways dictating graft rejection mechanisms. Curr Opin Organ Transplant (2016) 21(1):40–4. 10.1097/MOT.0000000000000263 26575853 PMC4701596

[26] Safinia N Afzali B Atalar K Lombardi G Lechler RI T-cell alloimmunity and chronic allograft dysfunction. Kidney Int Suppl (2010) 78(119):S2–12. 10.1038/ki.2010.416 21116312

[27] Everly MJ Rebellato LM Haisch CE Ozawa M Parker K Briley KP Incidence and impact of de novo donor-specific alloantibody in primary renal allografts. Transplantation (2013) 95(3):410–7. 10.1097/TP.0b013e31827d62e3 23380861

[28] Lodhi SA Lamb KE Meier-Kriesche HU Solid organ allograft survival improvement in the United States: the long-term does not mirror the dramatic short-term success. Am J Transplant (2011) 11(6):1226–35. 10.1111/j.1600-6143.2011.03539.x 21564524

[29] Loupy A Hill GS Jordan SC. The impact of donor-specific anti-HLA antibodies on late kidney allograft failure. Nat Rev Nephrol (2012) 8(6):348–57. 10.1038/nrneph.2012.81 22508180

[30] Jeannet M Pinn VW Flax MH Winn HJ Russell PS Humoral antibodies in renal allotransplantation in man. N Engl J Med (1970) 282(3):111–7. 10.1056/NEJM197001152820301 4902226

[31] Lee PC Terasaki PI Takemoto SK Lee PH Hung CJ Chen YL All chronic rejection failures of kidney transplants were preceded by the development of HLA antibodies. Transplantation (2002) 74(8):1192–4. 10.1097/00007890-200210270-00025 12438971

[32] Le Pavec J Suberbielle C Lamrani L Feuillet S Savale L Dorfmuller P De-novo donor-specific anti-HLA antibodies 30 days after lung transplantation are associated with a worse outcome. J Heart Lung Transplant (2016) 35(9):1067–77. 10.1016/j.healun.2016.05.020 27373824

[33] Tran A Fixler D Huang R Meza T Lacelle C Das BB. Donor-specific HLA alloantibodies: impact on cardiac allograft vasculopathy, rejection, and survival after pediatric heart transplantation. J Heart Lung Transplant (2016) 35(1):87–91. 10.1016/j.healun.2015.08.008 26422083

[34] Kaneku H O’Leary JG Banuelos N Jennings LW Susskind BM Klintmalm GB De novo donor-specific HLA antibodies decrease patient and graft survival in liver transplant recipients. Am J Transplant (2013) 13(6):1541–8. 10.1111/ajt.12212 23721554 PMC4408873

[35] Everly MJ Rebellato LM Haisch CE Briley KP Bolin P Kendrick WT Impact of IgM and IgG3 anti-HLA alloantibodies in primary renal allograft recipients. Transplantation (2014) 97(5):494–501. 10.1097/01.TP.0000441362.11232.48 24487396

[36] Arnold ML Heinemann FM Horn P Ziemann M Lachmann N Muhlbacher A 16(th) IHIW: anti-HLA alloantibodies of the of IgA isotype in re-transplant candidates. Int J Immunogenet (2013) 40(1):17–20. 10.1111/iji.12032 23280184

[37] Loupy A Lefaucheur C Vernerey D Prugger C Duong van Huyen JP Mooney N Complement-binding anti-HLA antibodies and kidney-allograft survival. N Engl J Med (2013) 369(13):1215–26. 10.1056/NEJMoa1302506 24066742

[38] Lefaucheur C Viglietti D Bentlejewski C Duong van Huyen JP Vernerey D Aubert O IgG donor-specific anti-human HLA antibody subclasses and kidney allograft antibody-mediated injury. J Am Soc Nephrol (2016) 27(1):293–304. 10.1681/ASN.2014111120 26293822 PMC4696574

[39] Thaunat O Patey N Caligiuri G Gautreau C Mamani-Matsuda M Mekki Y Chronic rejection triggers the development of an aggressive intragraft immune response through recapitulation of lymphoid organogenesis. J Immunol (2010) 185(1):717–28. 10.4049/jimmunol.0903589 20525884

[40] Martin L Guignier F Mousson C Rageot D Justrabo E Rifle G. Detection of donor-specific anti-HLA antibodies with flow cytometry in eluates and sera from renal transplant recipients with chronic allograft nephropathy. Transplantation (2003) 76(2):395–400. 10.1097/01.TP.0000078895.24606.45 12883199

[41] Martin L Charon-Barra C Bocrie O Guignier F D’Athis P Dautin G Detection of plasma cells, C4d deposits and donor-specific antibodies on sequential graft biopsies of renal transplant recipients with chronic dysfunction. Transpl Immunol (2010) 22(3–4):110–4. 10.1016/j.trim.2009.11.001 19900552

[42] Bachelet T Couzi L Lepreux S Legeret M Pariscoat G Guidicelli G Kidney intragraft donor-specific antibodies as determinant of antibody-mediated lesions and poor graft outcome. Am J Transplant (2013) 13(11):2855–64. 10.1111/ajt.12438 24102857

[43] Huibers MM Gareau AJ Beerthuijzen JM Siera-de Koning E van Kuik J Kamburova EG Donor-specific antibodies are produced locally in ectopic lymphoid structures in cardiac allografts. Am J Transplant (2016). 10.1111/ajt.13969 27428759

[44] Milongo D Kamar N Del Bello A Guilbeau-Frugier C Sallusto F Esposito L Allelic and epitopic characterization of intra-kidney-allograft anti-HLA antibodies at allograft nephrectomy. Am J Transplant (2016). 10.1111/ajt.13958 27402017

[45] Perry DK Pollinger HS Burns JM Rea D Ramos E Platt JL Two novel assays of alloantibody-secreting cells demonstrating resistance to desensitization with IVIG and rATG. Am J Transplant (2008) 8(1):133–43. 10.1111/j.1600-6143.2007.02039.x 18184311

[46] Lanzavecchia A Bernasconi N Traggiai E Ruprecht CR Corti D Sallusto F. Understanding and making use of human memory B cells. Immunol Rev (2006) 211:303–9. 10.1111/j.0105-2896.2006.00403.x 16824137 PMC7165660

[47] Tangye SG Avery DT Deenick EK Hodgkin PD. Intrinsic differences in the proliferation of naive and memory human B cells as a mechanism for enhanced secondary immune responses. J Immunol (2003) 170(2):686–94. 10.4049/jimmunol.170.2.686 12517929

[48] Tangye SG Avery DT Hodgkin PD A division-linked mechanism for the rapid generation of Ig-secreting cells from human memory B cells. J Immunol (2003) 170(1):261–9. 10.4049/jimmunol.170.1.261 12496408

[49] Han M Rogers J Lavingia B Stastny P. Peripheral blood B cells producing donor-specific HLA antibodies in vitro. Hum Immunol (2009) 70(1):29–34. 10.1016/j.humimm.2008.10.013 19026703

[50] Snanoudj R Claas FH Heidt S Legendre C Chatenoud L Candon S. Restricted specificity of peripheral alloreactive memory B cells in HLA-sensitized patients awaiting a kidney transplant. Kidney Int (2015) 87(6):1230–40. 10.1038/ki.2014.390 25565312

[51] Mulder A Eijsink C Kardol MJ Franke-van Dijk ME van der Burg SH Kester M Identification, isolation, and culture of HLA-A2-specific B lymphocytes using MHC class I tetramers. J Immunol (2003) 171(12):6599–603. 10.4049/jimmunol.171.12.6599 14662862

[52] Zachary AA Kopchaliiska D Montgomery RA Leffell MS. HLA-specific B cells: I. A method for their detection, quantification, and isolation using HLA tetramers. Transplantation (2007) 83(7):982–8. 10.1097/01.tp.0000259017.32857.99 17460571

[53] Zachary AA Kopchaliiska D Montgomery RA Melancon JK Leffell MS HLA-specific B cells: II. Application to transplantation. Transplantation (2007) 83(7):989–94. 10.1097/01.tp.0000259019.68244.d7 17460572

[54] Zachary AA Lucas DP Montgomery RA Leffell MS. Rituximab prevents an anamnestic response in patients with cryptic sensitization to HLA. Transplantation (2013) 95(5):701–4. 10.1097/TP.0b013e31827be3c1 23503502

[55] Heidt S Roelen DL de Vaal YJ Kester MG Eijsink C Thomas S A novel ELISPOT assay to quantify HLA-specific B cells in HLA-immunized individuals. Am J Transplant (2012) 12(6):1469–78. 10.1111/j.1600-6143.2011.03982.x 22390272

[56] Karahan GE de Vaal YJ Roelen DL Buchli R Claas FH Heidt S. Quantification of HLA class II-specific memory B cells in HLA-sensitized individuals. Hum Immunol (2015) 76(2–3):129–36. 10.1016/j.humimm.2015.01.014 25636565

[57] Lucia M Luque S Crespo E Melilli E Cruzado JM Martorell J Preformed circulating HLA-specific memory B cells predict high risk of humoral rejection in kidney transplantation. Kidney Int (2015) 88(4):874–87. 10.1038/ki.2015.205 26176829

[58] Constant S Schweitzer N West J Ranney P Bottomly K B lymphocytes can be competent antigen-presenting cells for priming CD4+ T cells to protein antigens in vivo. J Immunol (1995) 155(8):3734–41. 7561077

[59] Ron Y Sprent J T cell priming in vivo: a major role for B cells in presenting antigen to T cells in lymph nodes. J Immunol (1987) 138(9):2848–56. 2952725

[60] Wilson JL Cunningham AC Kirby JA. Alloantigen presentation by B cells: analysis of the requirement for B-cell activation. Immunology (1995) 86(3):325–30. 8550066 PMC1383932

[61] Janeway CA Jr Ron J Katz ME. The B cell is the initiating antigen-presenting cell in peripheral lymph nodes. J Immunol (1987) 138(4):1051–5. 3100626

[62] Constant SL B lymphocytes as antigen-presenting cells for CD4+ T cell priming in vivo. J Immunol (1999) 162(10):5695–703. 10229801

[63] Lanzavecchia A Pillars article: antigen-specific interaction between T and B cells. 1985. J Immunol (2007) 179(11):7206–8. 18025160

[64] Crawford A Macleod M Schumacher T Corlett L Gray D. Primary T cell expansion and differentiation in vivo requires antigen presentation by B cells. J Immunol (2006) 176(6):3498–506. 10.4049/jimmunol.176.6.3498 16517718

[65] Brandle D Joergensen J Zenke G Burki K Hof RP. Contribution of donor-specific antibodies to acute allograft rejection: evidence from B cell-deficient mice. Transplantation (1998) 65(11):1489–93. 10.1097/00007890-199806150-00014 9645808

[66] Epstein MM Di Rosa F Jankovic D Sher A Matzinger P. Successful T cell priming in B cell-deficient mice. J Exp Med (1995) 182(4):915–22. 10.1084/jem.182.4.915 7561694 PMC2192294

[67] Di Rosa F Matzinger P. Long-lasting CD8 T cell memory in the absence of CD4 T cells or B cells. J Exp Med (1996) 183(5):2153–63. 10.1084/jem.183.5.2153 8642325 PMC2192562

[68] Moulin V Andris F Thielemans K Maliszewski C Urbain J Moser M B lymphocytes regulate dendritic cell (DC) function in vivo: increased interleukin 12 production by DCs from B cell-deficient mice results in T helper cell type 1 deviation. J Exp Med (2000) 192(4):475–82. 10.1084/jem.192.4.475 10952717 PMC2193241

[69] Noorchashm H Reed AJ Rostami SY Mozaffari R Zekavat G Koeberlein B B cell-mediated antigen presentation is required for the pathogenesis of acute cardiac allograft rejection. J Immunol (2006) 177(11):7715–22. 10.4049/jimmunol.177.11.7715 17114442

[70] Ng YH Oberbarnscheidt MH Chandramoorthy HC Hoffman R Chalasani G B cells help alloreactive T cells differentiate into memory T cells. Am J Transplant (2010) 10(9):1970–80. 10.1111/j.1600-6143.2010.03223.x 20883532 PMC2956128

[71] Burns AM Ma L Li Y Yin D Shen J Xu J Memory alloreactive B cells and alloantibodies prevent anti-CD154-mediated allograft acceptance. J Immunol (2009) 182(3):1314–24. 10.4049/jimmunol.182.3.1314 19155477

[72] Sarwal M Chua MS Kambham N Hsieh SC Satterwhite T Masek M Molecular heterogeneity in acute renal allograft rejection identified by DNA microarray profiling. N Engl J Med (2003) 349(2):125–38. 10.1056/NEJMoa035588 12853585

[73] Hippen BE DeMattos A Cook WJ Kew CE II Gaston RS. Association of CD20+ infiltrates with poorer clinical outcomes in acute cellular rejection of renal allografts. Am J Transplant (2005) 5(9):2248–52. 10.1111/j.1600-6143.2005.01009.x 16095505

[74] Tsai EW Rianthavorn P Gjertson DW Wallace WD Reed EF Ettenger RB. CD20+ lymphocytes in renal allografts are associated with poor graft survival in pediatric patients. Transplantation (2006) 82(12):1769–73. 10.1097/01.tp.0000250572.46679.45 17198274

[75] Zarkhin V Kambham N Li L Kwok S Hsieh SC Salvatierra O Characterization of intra-graft B cells during renal allograft rejection. Kidney Int (2008) 74(5):664–73. 10.1038/ki.2008.249 18547992

[76] Kayler LK Lakkis FG Morgan C Basu A Blisard D Tan HP Acute cellular rejection with CD20-positive lymphoid clusters in kidney transplant patients following lymphocyte depletion. Am J Transplant (2007) 7(4):949–54. 10.1111/j.1600-6143.2007.01737.x 17331114

[77] Doria C di Francesco F Ramirez CB Frank A Iaria M Francos G The presence of B-cell nodules does not necessarily portend a less favorable outcome to therapy in patients with acute cellular rejection of a renal allograft. Transplant Proc (2006) 38(10):3441–4. 10.1016/j.transproceed.2006.10.173 17175297

[78] Bagnasco SM Tsai W Rahman MH Kraus ES Barisoni L Vega R CD20-positive infiltrates in renal allograft biopsies with acute cellular rejection are not associated with worse graft survival. Am J Transplant (2007) 7(8):1968–73. 10.1111/j.1600-6143.2007.01885.x 17617861

[79] Liarski VM Kaverina N Chang A Brandt D Yanez D Talasnik L Cell distance mapping identifies functional T follicular helper cells in inflamed human renal tissue. Sci Transl Med (2014) 6(230):230ra46. 10.1126/scitranslmed.3008146 24695686 PMC4129446

[80] Aloisi F Pujol-Borrell R. Lymphoid neogenesis in chronic inflammatory diseases. Nat Rev Immunol (2006) 6(3):205–17. 10.1038/nri1786 16498451

[81] Figenschau SL Fismen S Fenton KA Fenton C Mortensen ES. Tertiary lymphoid structures are associated with higher tumor grade in primary operable breast cancer patients. BMC Cancer (2015) 15:101. 10.1186/s12885-015-1116-1 25884667 PMC4357183

[82] Hsiao HM Li W Gelman AE Krupnick AS Kreisel D. The role of lymphoid neogenesis in allografts. Am J Transplant (2016) 16(4):1079–85. 10.1111/ajt.13645 26614734 PMC4803576

[83] Kerjaschki D Regele HM Moosberger I Nagy-Bojarski K Watschinger B Soleiman A Lymphatic neoangiogenesis in human kidney transplants is associated with immunologically active lymphocytic infiltrates. J Am Soc Nephrol (2004) 15(3):603–12. 10.1097/01.ASN.0000113316.52371.2E 14978162

[84] Thaunat O Field AC Dai J Louedec L Patey N Bloch MF Lymphoid neogenesis in chronic rejection: evidence for a local humoral alloimmune response. Proc Natl Acad Sci U S A (2005) 102(41):14723–8. 10.1073/pnas.0507223102 16192350 PMC1253595

[85] Cheng J Torkamani A Grover RK Jones TM Ruiz DI Schork NJ Ectopic B-cell clusters that infiltrate transplanted human kidneys are clonal. Proc Natl Acad Sci U S A (2011) 108(14):5560–5. 10.1073/pnas.1101148108 21415369 PMC3078383

[86] Adair A Mitchell DR Kipari T Qi F Bellamy CO Robertson F Peritubular capillary rarefaction and lymphangiogenesis in chronic allograft failure. Transplantation (2007) 83(12):1542–50. 10.1097/01.tp.0000266689.93615.cd 17589335

[87] Fillatreau S Sweenie CH McGeachy MJ Gray D Anderton SM B cells regulate autoimmunity by provision of IL-10. Nat Immunol (2002) 3(10):944–50. 10.1038/ni833 12244307

[88] Matsushita T Yanaba K Bouaziz JD Fujimoto M Tedder TF. Regulatory B cells inhibit EAE initiation in mice while other B cells promote disease progression. J Clin Invest (2008) 118(10):3420–30. 10.1172/JCI36030 18802481 PMC2542851

[89] Blair PA Norena LY Flores-Borja F Rawlings DJ Isenberg DA Ehrenstein MR CD19(+)CD24(hi)CD38(hi) B cells exhibit regulatory capacity in healthy individuals but are functionally impaired in systemic lupus erythematosus patients. Immunity (2010) 32(1):129–40. 10.1016/j.immuni.2009.11.009 20079667

[90] Duddy M Niino M Adatia F Hebert S Freedman M Atkins H Distinct effector cytokine profiles of memory and naive human B cell subsets and implication in multiple sclerosis. J Immunol (2007) 178(10):6092–9. 10.4049/jimmunol.178.10.6092 17475834

[91] Matsumoto M Baba A Yokota T Nishikawa H Ohkawa Y Kayama H Interleukin-10-producing plasmablasts exert regulatory function in autoimmune inflammation. Immunity (2014) 41(6):1040–51. 10.1016/j.immuni.2014.10.016 25484301

[92] Shen P Roch T Lampropoulou V O’Connor RA Stervbo U Hilgenberg E IL-35-producing B cells are critical regulators of immunity during autoimmune and infectious diseases. Nature (2014) 507(7492):366–70. 10.1038/nature12979 24572363 PMC4260166

[93] Lee KM Stott RT Zhao G SooHoo J Xiong W Lian MM TGF-beta-producing regulatory B cells induce regulatory T cells and promote transplantation tolerance. Eur J Immunol (2014) 44(6):1728–36. 10.1002/eji.201344062 24700192 PMC4048633

[94] Ding Q Yeung M Camirand G Zeng Q Akiba H Yagita H Regulatory B cells are identified by expression of TIM-1 and can be induced through TIM-1 ligation to promote tolerance in mice. J Clin Invest (2011) 121(9):3645–56. 10.1172/JCI46274 21821911 PMC3163958

[95] Iwata Y Matsushita T Horikawa M Dilillo DJ Yanaba K Venturi GM Characterization of a rare IL-10-competent B-cell subset in humans that parallels mouse regulatory B10 cells. Blood (2011) 117(2):530–41. 10.1182/blood-2010-07-294249 20962324 PMC3031478

[96] Lee KM Kim JI Stott R Soohoo J O’Connor MR Yeh H Anti-CD45RB/anti-TIM-1-induced tolerance requires regulatory B cells. Am J Transplant (2012) 12(8):2072–8. 10.1111/j.1600-6143.2012.04055.x 22494812 PMC3396747

[97] Le Texier L Thebault P Lavault A Usal C Merieau E Quillard T Long-term allograft tolerance is characterized by the accumulation of B cells exhibiting an inhibited profile. Am J Transplant (2011) 11(3):429–38. 10.1111/j.1600-6143.2010.03336.x 21114655

[98] Newell KA Asare A Kirk AD Gisler TD Bourcier K Suthanthiran M Identification of a B cell signature associated with renal transplant tolerance in humans. J Clin Invest (2010) 120(6):1836–47. 10.1172/JCI39933 20501946 PMC2877933

[99] Sagoo P Perucha E Sawitzki B Tomiuk S Stephens DA Miqueu P Development of a cross-platform biomarker signature to detect renal transplant tolerance in humans. J Clin Invest (2010) 120(6):1848–61. 10.1172/JCI39922 20501943 PMC2877932

[100] Heidt S Wood KJ. Biomarkers of operational tolerance in solid organ transplantation. Expert Opin Med Diagn (2012) 6(4):281–93. 10.1517/17530059.2012.680019 22988481 PMC3442251

[101] Viklicky O Krystufkova E Brabcova I Sekerkova A Wohlfahrt P Hribova P B-cell-related biomarkers of tolerance are up-regulated in rejection-free kidney transplant recipients. Transplantation (2013) 95(1):148–54. 10.1097/TP.0b013e3182789a24 23222918

[102] Heidt S Vergunst M Anholts JD Reinders ME de Fijter JW Eikmans M B cell markers of operational tolerance can discriminate acute kidney allograft rejection from stable graft function. Transplantation (2015) 99(5):1058–64. 10.1097/TP.0000000000000465 25340606

[103] Pallier A Hillion S Danger R Giral M Racape M Degauque N Patients with drug-free long-term graft function display increased numbers of peripheral B cells with a memory and inhibitory phenotype. Kidney Int (2010) 78(5):503–13. 10.1038/ki.2010.162 20531452

[104] Chesneau M Pallier A Braza F Lacombe G Le Gallou S Baron D Unique B cell differentiation profile in tolerant kidney transplant patients. Am J Transplant (2014) 14(1):144–55. 10.1111/ajt.12508 24354874

[105] Chesneau M Michel L Dugast E Chenouard A Baron D Pallier A Tolerant kidney transplant patients produce B cells with regulatory properties. J Am Soc Nephrol (2015) 26(10):2588–98. 10.1681/ASN.2014040404 25644114 PMC4587683

[106] Ekberg H Tedesco-Silva H Demirbas A Vitko S Nashan B Gurkan A Reduced exposure to calcineurin inhibitors in renal transplantation. N Engl J Med (2007) 357(25):2562–75. 10.1056/NEJMoa067411 18094377

[107] Heidt S Roelen DL Eijsink C Eikmans M van Kooten C Claas FH Calcineurin inhibitors affect B cell antibody responses indirectly by interfering with T cell help. Clin Exp Immunol (2010) 159(2):199–207. 10.1111/j.1365-2249.2009.04051.x 19922499 PMC2810388

[108] Heidt S Roelen DL Eijsink C van Kooten C Claas FH Mulder A. Effects of immunosuppressive drugs on purified human B cells: evidence supporting the use of MMF and rapamycin. Transplantation (2008) 86(9):1292–300. 10.1097/TP.0b013e3181874a36 19005412

[109] De Bruyne R Bogaert D De Ruyck N Lambrecht BN Van Winckel M Gevaert P Calcineurin inhibitors dampen humoral immunity by acting directly on naive B cells. Clin Exp Immunol (2015) 180(3):542–50. 10.1111/cei.12604 25682989 PMC4449782

[110] Jordan SC Pescovitz MD. Presensitization: the problem and its management. Clin J Am Soc Nephrol (2006) 1(3):421–32. 10.2215/CJN.01651105 17699241

[111] Donauer J Wilpert J Geyer M Schwertfeger E Kirste G Drognitz O ABO-incompatible kidney transplantation using antigen-specific immunoadsorption and rituximab: a single center experience. Xenotransplantation (2006) 13(2):108–10. 10.1111/j.1399-3089.2006.00293.x 16623802

[112] Tyden G Donauer J Wadstrom J Kumlien G Wilpert J Nilsson T Implementation of a Protocol for ABO-incompatible kidney transplantation – a three-center experience with 60 consecutive transplantations. Transplantation (2007) 83(9):1153–5. 10.1097/01.tp.0000262570.18117.55 17496528

[113] Kahwaji J Sinha A Toyoda M Ge S Reinsmoen N Cao K Infectious complications in kidney-transplant recipients desensitized with rituximab and intravenous immunoglobulin. Clin J Am Soc Nephrol (2011) 6(12):2894–900. 10.2215/CJN.03710411 22157713 PMC3255382

[114] Clatworthy MR Watson CJ Plotnek G Bardsley V Chaudhry AN Bradley JA B-cell-depleting induction therapy and acute cellular rejection. N Engl J Med (2009) 360(25):2683–5. 10.1056/NEJMc0808481 19535812 PMC4143588

[115] Tyden G Genberg H Tollemar J Ekberg H Persson NH Tufveson G A randomized, doubleblind, placebo-controlled, study of single-dose rituximab as induction in renal transplantation. Transplantation (2009) 87(9):1325–9. 10.1097/TP.0b013e3181a235fd 19424032

[116] Becker YT Becker BN Pirsch JD Sollinger HW. Rituximab as treatment for refractory kidney transplant rejection. Am J Transplant (2004) 4(6):996–1001. 10.1111/j.1600-6143.2004.00454.x 15147435

[117] Alausa M Almagro U Siddiqi N Zuiderweg R Medipalli R Hariharan S. Refractory acute kidney transplant rejection with CD20 graft infiltrates and successful therapy with rituximab. Clin Transplant (2005) 19(1):137–40. 10.1111/j.1399-0012.2004.00292.x 15659147

[118] Lehnhardt A Mengel M Pape L Ehrich JH Offner G Strehlau J. Nodular B-cell aggregates associated with treatment refractory renal transplant rejection resolved by rituximab. Am J Transplant (2006) 6(4):847–51. 10.1111/j.1600-6143.2006.01246.x 16539643

[119] Steinmetz OM Lange-Husken F Turner JE Vernauer A Helmchen U Stahl RA Rituximab removes intrarenal B cell clusters in patients with renal vascular allograft rejection. Transplantation (2007) 84(7):842–50. 10.1097/01.tp.0000282786.58754.2b 17984836

[120] Zarkhin V Li L Kambham N Sigdel T Salvatierra O Sarwal MM A randomized, prospective trial of rituximab for acute rejection in pediatric renal transplantation. Am J Transplant (2008) 8(12):2607–17. 10.1111/j.1600-6143.2008.02411.x 18808404

[121] Thaunat O Patey N Gautreau C Lechaton S Fremeaux-Bacchi V Dieu-Nosjean MC B cell survival in intragraft tertiary lymphoid organs after rituximab therapy. Transplantation (2008) 85(11):1648–53. 10.1097/TP.0b013e3181735723 18551073

[122] Kamburova EG Koenen HJ van den Hoogen MW Baas MC Joosten I Hilbrands LB. Longitudinal analysis of T and B cell phenotype and function in renal transplant recipients with or without rituximab induction therapy. PLoS One (2014) 9(11):e112658. 10.1371/journal.pone.0112658 25393622 PMC4231065

[123] Heidt S Hester J Shankar S Friend PJ Wood KJ B-cell repopulation after alemtuzumab induction-transient increase in transitional B cells and long-term dominance of naive B cells. Am J Transplant (2012) 12(7):1784–92. 10.1111/j.1600-6143.2012.04012.x 22420490 PMC3387484

[124] Cherukuri A Salama AD Carter C Smalle N McCurtin R Hewitt EW An analysis of lymphocyte phenotype after steroid avoidance with either alemtuzumab or basiliximab induction in renal transplantation. Am J Transplant (2012) 12(4):919–31. 10.1111/j.1600-6143.2011.03891.x 22390816

[125] Chen J Wang Q Yin D Vu V Sciammas R Chong AS. Cutting edge: CTLA-4Ig inhibits memory b cell responses and promotes allograft survival in sensitized recipients. J Immunol (2015) 195(9):4069–73. 10.4049/jimmunol.1500940 26416270 PMC4610858

[126] Kim EJ Kwun J Gibby AC Hong JJ Farris AB III Iwakoshi NN Costimulation blockade alters germinal center responses and prevents antibody-mediated rejection. Am J Transplant (2014) 14(1):59–69. 10.1111/ajt.12526 24354871 PMC3985346

[127] Vincenti F Rostaing L Grinyo J Rice K Steinberg S Gaite L Belatacept and long-term outcomes in kidney transplantation. N Engl J Med (2016) 374(4):333–43. 10.1056/NEJMoa1506027 26816011

[128] Bloom D Chang Z Pauly K Kwun J Fechner J Hayes C BAFF is increased in renal transplant patients following treatment with alemtuzumab. Am J Transplant (2009) 9(8):1835–45. 10.1111/j.1600-6143.2009.02710.x 19522878 PMC4876605

[129] Zarkhin V Li L Sarwal MM BAFF may modulate the rate of B-cell repopulation after rituximab therapy for acute renal transplant rejection. Transplantation (2009) 88(10):1229–30. 10.1097/TP.0b013e3181bbba1a 19935379

[130] Mackay F Schneider P. Cracking the BAFF code. Nat Rev Immunol (2009) 9(7):491–502. 10.1038/nri2572 19521398

[131] Thibault-Espitia A Foucher Y Danger R Migone T Pallier A Castagnet S BAFF and BAFF-R levels are associated with risk of long-term kidney graft dysfunction and development of donor-specific antibodies. Am J Transplant (2012) 12(10):2754–62. 10.1111/j.1600-6143.2012.04194.x 22883025

[132] Banham G Prezzi D Harford S Taylor CJ Hamer R Higgins R Elevated pretransplantation soluble BAFF is associated with an increased risk of acute antibody-mediated rejection. Transplantation (2013) 96(4):413–20. 10.1097/TP.0b013e318298dd65 23842189 PMC4170143

[133] Snanoudj R Candon S Roelen DL Jais JP Claas FH Legendre C Peripheral B-cell phenotype and BAFF levels are associated with HLA immunization in patients awaiting kidney transplantation. Transplantation (2014) 97(9):917–24. 10.1097/01.TP.0000438211.34842.5e 24827764

[134] Ye Q Wang L Wells AD Tao R Han R Davidson A BAFF binding to T cell-expressed BAFF-R costimulates T cell proliferation and alloresponses. Eur J Immunol (2004) 34(10):2750–9. 10.1002/eji.200425198 15368291

[135] Kwun J Page E Hong JJ Gibby A Yoon J Farris AB Neutralizing BAFF/APRIL with atacicept prevents early DSA formation and AMR development in T cell depletion induced nonhuman primate AMR model. Am J Transplant (2015) 15(3):815–22. 10.1111/ajt.13045 25675879 PMC5504528

[136] Manasanch EE Korde N Zingone A Tageja N Fernandez de Larrea C Bhutani M The proteasome: mechanisms of biology and markers of activity and response to treatment in multiple myeloma. Leuk Lymphoma (2014) 55(8):1707–14. 10.3109/10428194.2013.828351 24261677

[137] Everly MJ Terasaki PI Hopfield J Trivedi HL Kaneku H. Protective immunity remains intact after antibody removal by means of proteasome inhibition. Transplantation (2010) 90(12):1493–8. 10.1097/TP.0b013e3181ff87b1 21042236

[138] Perry DK Burns JM Pollinger HS Amiot BP Gloor JM Gores GJ Proteasome inhibition causes apoptosis of normal human plasma cells preventing alloantibody production. Am J Transplant (2009) 9(1):201–9. 10.1111/j.1600-6143.2008.02461.x 18976291

[139] Everly MJ Everly JJ Susskind B Brailey P Arend LJ Alloway RR Bortezomib provides effective therapy for antibody- and cell-mediated acute rejection. Transplantation (2008) 86(12):1754–61. 10.1097/TP.0b013e318190af83 19104417

[140] Woodle ES Shields AR Ejaz NS Sadaka B Girnita A Walsh RC Prospective iterative trial of proteasome inhibitor-based desensitization. Am J Transplant (2015) 15(1):101–18. 10.1111/ajt.13050 25534446

[141] Mulder A Heidt S Vergunst M Roelen DL Claas FH. Proteasome inhibition profoundly affects activated human B cells. Transplantation (2013) 95(11):1331–7. 10.1097/TP.0b013e3182911739 23624544

